# Structural Brain Network Changes across the Adult Lifespan

**DOI:** 10.3389/fnagi.2017.00275

**Published:** 2017-08-17

**Authors:** Ke Liu, Shixiu Yao, Kewei Chen, Jiacai Zhang, Li Yao, Ke Li, Zhen Jin, Xiaojuan Guo

**Affiliations:** ^1^College of Information Science and Technology, Beijing Normal University Beijing, China; ^2^Banner Alzheimer's Institute Phoenix, AZ, United States; ^3^Department of Mathematics and Statistics, Arizona State University Tempe, AZ, United States; ^4^Arizona Alzheimer's Consortium Phoenix, AZ, United States; ^5^National Key Laboratory of Cognitive Neuroscience and Learning, Beijing Normal University Beijing, China; ^6^Laboratory of Magnetic Resonance Imaging, Beijing 306 Hospital Beijing, China; ^7^Beijing Key Laboratory of Brain Imaging and Connectomics, Beijing Normal University Beijing, China

**Keywords:** independent component analysis, structural network, magnetic resonance imaging, gray matter volume, age-related changes

## Abstract

A number of magnetic resonance imaging (MRI) studies have shown age-related alterations in brain structural networks in different age groups. However, the specific age-associated changes in brain structural networks across the adult lifespan is underexplored. In the current study, we performed a multivariate independent component analysis (ICA) to identify structural brain networks based on covariant gray matter volume and then investigated the age-related trajectories of structural networks over the adult lifespan in 536 healthy subjects aged 20–86 years. Twenty independent components (ICs) were extracted in the ICA, and statistical analyses between age and ICA weights revealed 16 age-related ICs across the adult lifespan. Most of the trajectories of ICA weights demonstrated significant linear decline tendencies, and the corresponding structural networks primarily included the anterior and posterior dorsal attention networks, the ventral and posterior default mode networks, the auditory network, five cerebellum networks and the hippocampus-related network with the most significant decreased tendency among all ICs (*p* of age = 1.11E-77). Only the temporal lobe-related network showed a significant quadratic tendency with age (*p* of age^2^ = 5.66E-06). Our findings not only provide insight into the patterns of the age-related changes of structural networks but also provide a foundation for understanding abnormal aging.

## Introduction

Magnetic resonance imaging (MRI) studies have shown that the brain undergoes remarkable structural development during childhood and adolescence and that those alterations continue even through adulthood (Good et al., 2001; Gogtay et al., 2004; Raji et al., 2012; Fjell et al., 2013; Mills et al., 2014). The global gray matter volume decreases linearly with age, and the total white matter volume shows an inverse U-shaped tendency in healthy adults (Good et al., 2001; Ge et al., 2002); however, regional brain changes are heterogeneous in different regions across the adult lifespan (Gogtay et al., 2004; Allen et al., 2005; Curiati et al., 2009; Ziegler et al., 2012; Fjell et al., 2013). For example, the gray matter volumes of the frontal, parietal and occipital lobes present linear decreases with age across the adult lifespan (Allen et al., 2005), while the hippocampus volume presents a non-linear trend with age (Allen et al., 2005; Fjell et al., 2013). Early structural MRI researches used univariate methods, such as regions of interest (ROIs) or voxel-based morphometry (VBM), to investigate the age-related gray matter changes; however, these studies considered ROIs or brain voxels as independent variables and ignored the interregional covariant information among them.

A number of brain MRI studies have investigated anatomical network changes based on the structural covariance of gray matter volume in normal adults (Brickman et al., 2007; Bergfield et al., 2010; Li et al., 2013; Hafkemeijer et al., 2014). Li et al. used seed-ROI regression models to explore age-related changes of gray matter volumes in eight gray matter networks in young, middle-aged and older groups of healthy subjects aged 18–89 years (Li et al., 2013). Hafkemeijer et al. utilized independent component analysis (ICA) to extract nine gray matter anatomical networks in middle-aged to older normal participants (45–85 years), who were divided into four age subgroups (Hafkemeijer et al., 2014). Brickman et al. identified aging-related regional MRI covariance patterns in younger and older groups of healthy adults using a multivariate statistical model called the subprofile scaling model (SSM; Brickman et al., 2007). These studies showed that structural covariance patterns or networks demonstrated different age-related changes among the different age groups (Brickman et al., 2007; Li et al., 2013; Hafkemeijer et al., 2014). For example, there was a negative correlation between age and gray matter volume in four anatomical networks, including the medial visual cortical network, sensorimotor network, default mode network (DMN) and executive control network; however, gray matter volume was not significantly associated with age in five other networks, including the temporal network, auditory network, and three cerebellar networks (Hafkemeijer et al., 2014). It has been noted that the above-mentioned studies focused primarily on brain structural network changes in different age groups. However, the age-related trajectories of the brain structural networks across the adult lifespan need to be further explored.

Multivariate analysis methods can identify the inter-regional covariance relationship among different brain regions. Therefore, these approaches have been widely applied to brain imaging studies (Damoiseaux et al., 2006; Brickman et al., 2007; Mantini et al., 2007; Xu et al., 2009; Bergfield et al., 2010; McIntosh and Mišic, 2013; Guo et al., 2014; Hafkemeijer et al., 2014). ICA, as a popular data-driven multivariate analysis method, was introduced first to the studies of brain functional networks (Damoiseaux et al., 2006; Mantini et al., 2007) and then to those of structural networks (Xu et al., 2009; Guo et al., 2014; Hafkemeijer et al., 2014). Xu et al. presented a source-based morphometry (SBM) approach using ICA to study gray matter network differences between subjects with schizophrenia and healthy control subjects and confirmed the validity of ICA in structural MRI data (Xu et al., 2009). Guo et al. also applied ICA to examine structural covariance networks across healthy young adults and to determine their spatial consistency (Guo et al., 2014). Compared with other multivariate analysis methods, ICA is a higher-order statistical method and can decompose linear mixed signals into maximally independent components (Calhoun et al., 2009). In this way, ICA can effectively extract independent sources from complex brain imaging data without a priori information.

The purpose of the current study is to explore age-related gray matter changes at the network level across the adult lifespan. We applied the ICA method to identify structural gray matter covariance networks among 536 healthy subjects aged 20–86 years. Finally, regression analyses were performed on ICA weights and age to investigate age trajectories of the corresponding networks.

## Materials and methods

### Participants

Structural MRI data were obtained from a large public database of the Information eXtraction from Images (IXI) (http://brain-development.org/ixi-dataset/). In this study, 536 healthy subjects (Females/Males = 273/263, age range 20–86 years) were included. Specific information about the subjects is presented in Table 1. More details about demographic information of the participants are presented on the IXI database website (Kennedy et al., 2016).

**Table 1:** Sample characteristics of different age groups.

**Age span (yr)**	**Mean age (yr)**	**No. subjects**	**No. subjects in scanning site^*a*^**	**Female/male**	**Ethnicity^*b*^**	**Education mean^*c*^**
20–29	25.41	93	35/41/17	52/41	64/2/12/15	4.39
30–39	34.36	106	55/28/23	42/64	80/3/12/11	4.58
40–49	44.41	86	51/25/10	46/40	73/1/6/6	4.05
50–59	55.2	88	55/29/4	53/35	72/4/7/5	3.53
60–69	64.01	116	68/41/7	72/44	94/3/11/8	3.55
70–79	72.81	41	21/14/6	6/35	35/1/2/3	3.07
80–86	83.75	6	4/1/1	2/4	6/0/0/0	3.67
Total		536	289/179/68	273/263	424/14/50/48	3.94

a Three separate subsamples from different scanning sites in London: Guy's Hospital: Philips 1.5T/Hammersmith Hospital: Philips 3T/Institute of Psychiatry: General Electric 1.5T

b The number of different ethnic groups in our IXI sample: Caucasian/Black/Asian/Other

c *Education levels: 1 = No qualifications; 2 = O-levels, GCSEs, or CSE; 3 = A-levels; 4 = Further education; 5 = University or Polytechnic degrees*.

### Data acquisition

All structural MRIs were obtained on three different sites: a Philips 1.5T system at Guy's Hospital, a General Electric (GE) 1.5T at the Institute of Psychiatry, and a Philips 3T magnetic resonance scanner at Hammersmith Hospital. The T1-weighted structural MRIs were acquired using a magnetization-prepared rapid acquisition gradient-echo (MPRAGE) sequence. Scanning parameters for Philips 1.5T scanner were: TR = 9.8 ms, TE = 4.6 ms, flip angle = 8°; and for Philips 3T scanner were: TR = 9.6 ms, TE = 4.6 ms, flip angle = 8°. Scanning parameters for GE 1.5T scanner were not available.

### Image preprocessing

In this study, all structural MRI data were processed using the VBM8 toolbox (available at http://dbm.neuro.uni-jena.de/vbm8) (Ashburner and Friston, 2000; Good et al., 2001; Ashburner, 2007) in the Statistical Parametric Mapping (SPM8) software (available at: http://www.fil.ion.ucl.ac.uk/spm). In brief, using adaptive maximum posterior (MAP) and partial volume estimation (PVE), all of the structural images were segmented into gray matter, white matter and cerebrospinal fluid. Subsequently, a diffeomorphic anatomical registration exponential Lie algebra (DARTEL) approach (Ashburner, 2007) was applied to normalize each subject's gray matter image to the average DARTEL template, which was generated iteratively and finally to the Montreal Neurological Institute (MNI) space. Additionally, to preserve the total gray matter amount in the native space, the voxel of each gray matter image was multiplied by the Jacobian determinant from the normalization. Gaussian smoothing was performed with a kernel of 8 mm full width at half maximum (FWHM) on each subject's gray matter image.

Multiple linear regression models were constructed for the spatial preprocessed gray matter maps to account for two confounding factors: scanner and gender. In order to avoid the possible bias of different scanners, all participants from the three scanners (Guy's Hospital, Institute of Psychiatry and Hammersmith Hospital) were represented with three column dummy independent variables of 0/1 in regression models. Additionally, gender was a nuisance factor in this study, then gender was also represented with one column dummy variables of 0/1. The adjusted gray matter images were entered into the subsequent ICA procedure.

### ICA

The ICA was implemented using the fusion ICA toolbox (FIT) (available at http://mialab.mrn.org/software/fit/index.html). In this study, the gray matter image of each subject was spatially concatenated as a row vector to form a subject-by-voxel input data matrix. Then, the initial matrix was decomposed into a subject-by-source mixing matrix (also referred to as ICA weights) and a statistically independent source-by-voxel source matrix (spatial components or brain networks) using the informax algorithm which minimizes the mutual information of the sources (Calhoun et al., 2009; Xu et al., 2009). The mixing matrix exhibits the interrelationship in subjects and source networks, and the source matrix exhibits the interrelationship in source networks and voxels across the whole brain. Then each column of the mixing matrix represents the degree to which one subject contributes to the corresponding source network. Each row of the source matrix indicates a spatial distribution of brain structural network which expresses the covariant changes of the gray matter volume within the brain (Xu et al., 2009). Finally, each source network was converted to a z-score map and reshaped to a 3D brain map with a threshold *Z* = 3 to reveal the gray matter structural covariant patterns. The resulting ICA coefficient weights were used for the statistical analysis.

### Statistical analysis

Cubic, quadratic and linear regression analyses were performed separately between age (independent variables) and each column of the ICA weights (dependent variables) to explore the age-related trajectories of networks throughout the adult lifespan. Bayesian Information Criterion (BIC) was used to determine the optimal regression model with the smallest BIC value. A single-sample *T*-test was performed on the regression coefficients of the highest-order age item with the statistical significance threshold set at *p* < 0.05 with Bonferroni correction for each optimal regression model.

Additionally, in order to evaluate the age range effect on the age-related patterns, we re-performed the same statistical analysis of the ICs for subjects aged 20–80 years, 20–70 years, and 20–60 years, respectively.

## Results

**Figure 1:**
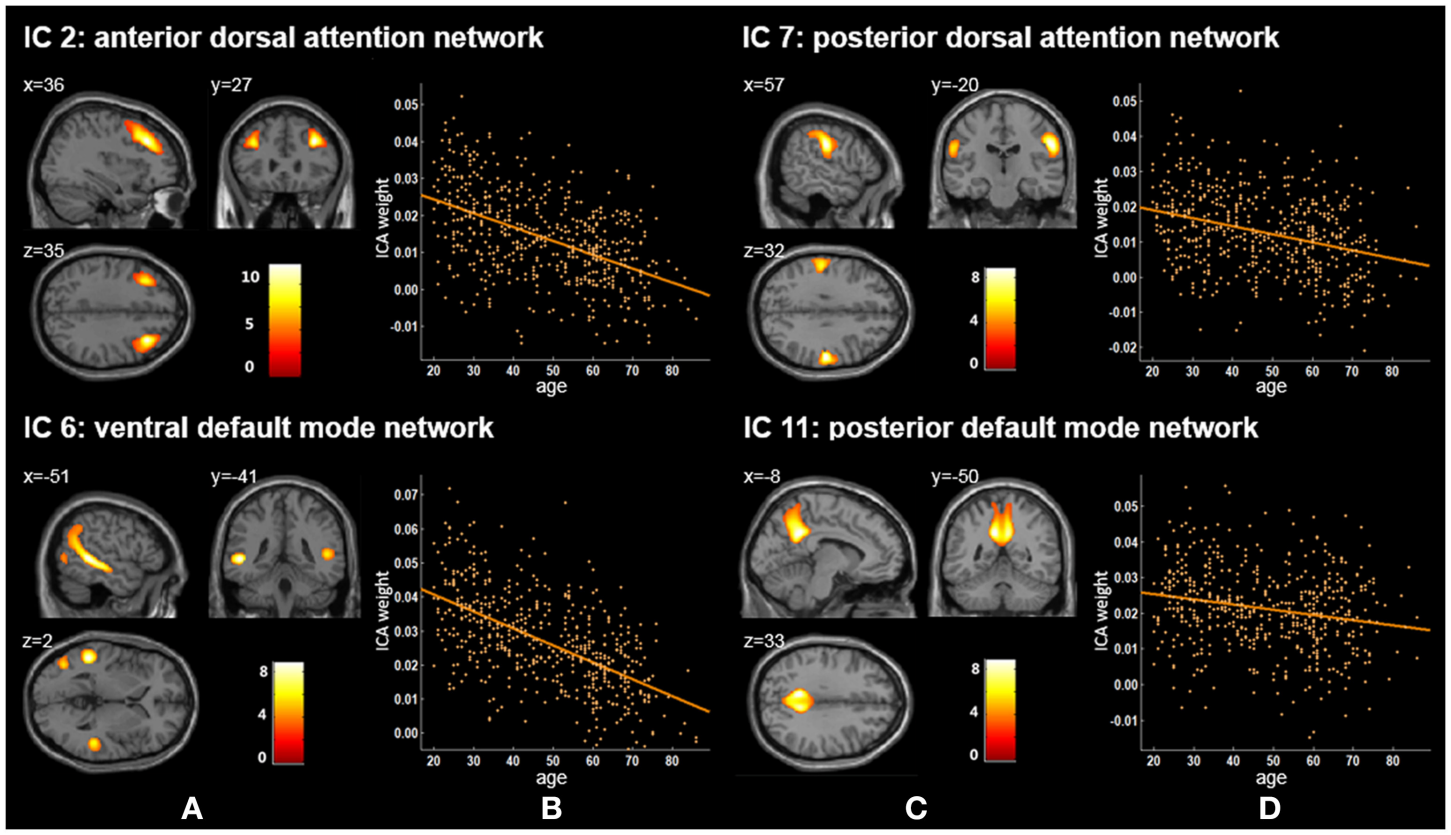
Age-related changes in gray matter structural networks. **(A)** and **(C)** columns: IC 2, 7, 6, and 11 represent structural network maps associated with age in 536 healthy adult subjects. The color bar represents Z scores. **(B)** and **(D)** columns: the orange scatterplots show the age-related patterns in different networks. The orange lines represent the fitted lines between age and ICA weights for each network.

**Figure 2:**
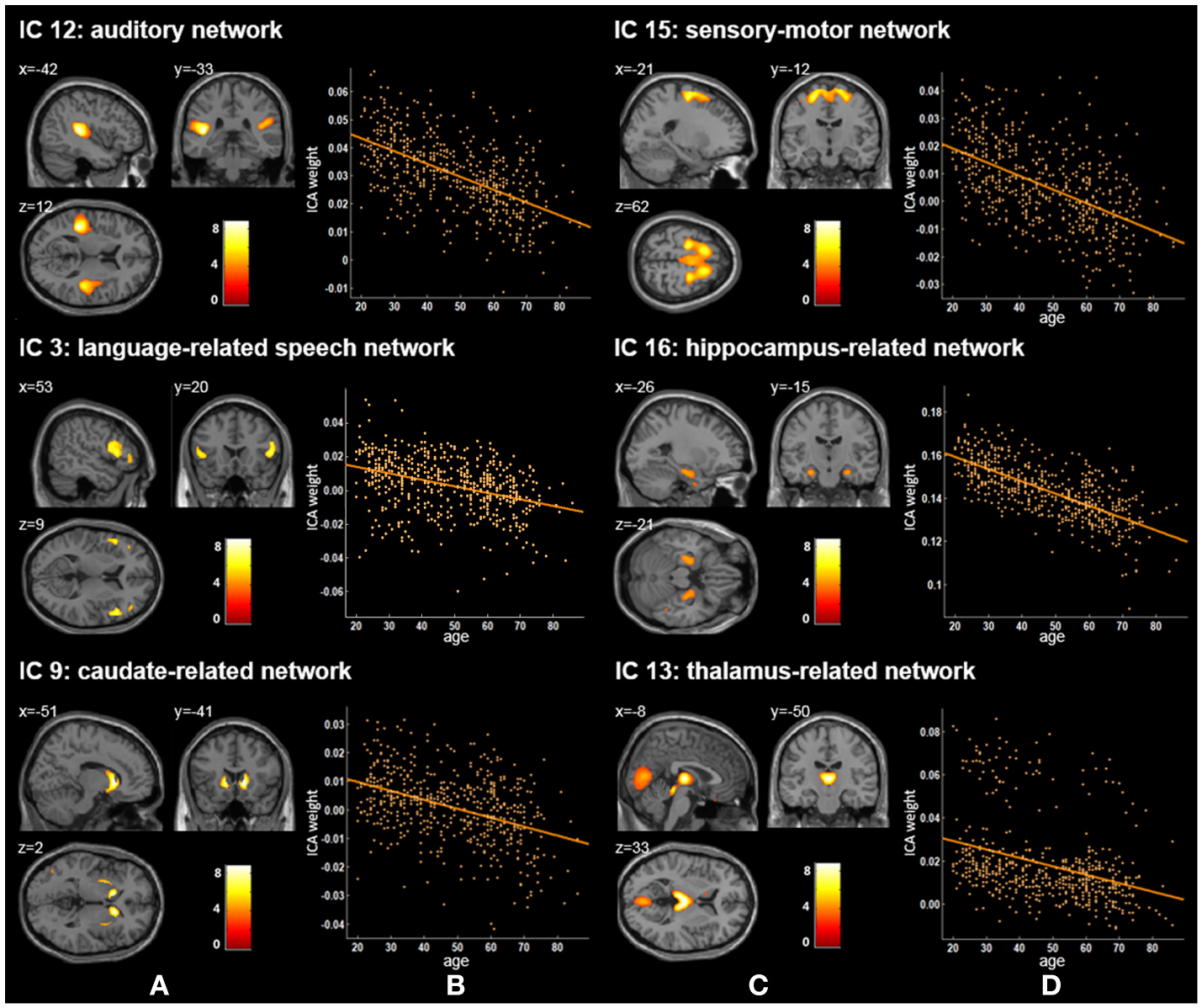
Age-related changes in gray matter structural networks. **(A)** and **(C)** columns: IC 12, 15, 3, 16, 9, and 13 represent structural network maps associated with age in 536 healthy adult subjects. The color bar represents Z scores. **(B)** and **(D)** column: the orange scatterplots show the age-related patterns in different networks. The orange lines represent the fitted lines between age and ICA weights for each network.

**Figure 3:**
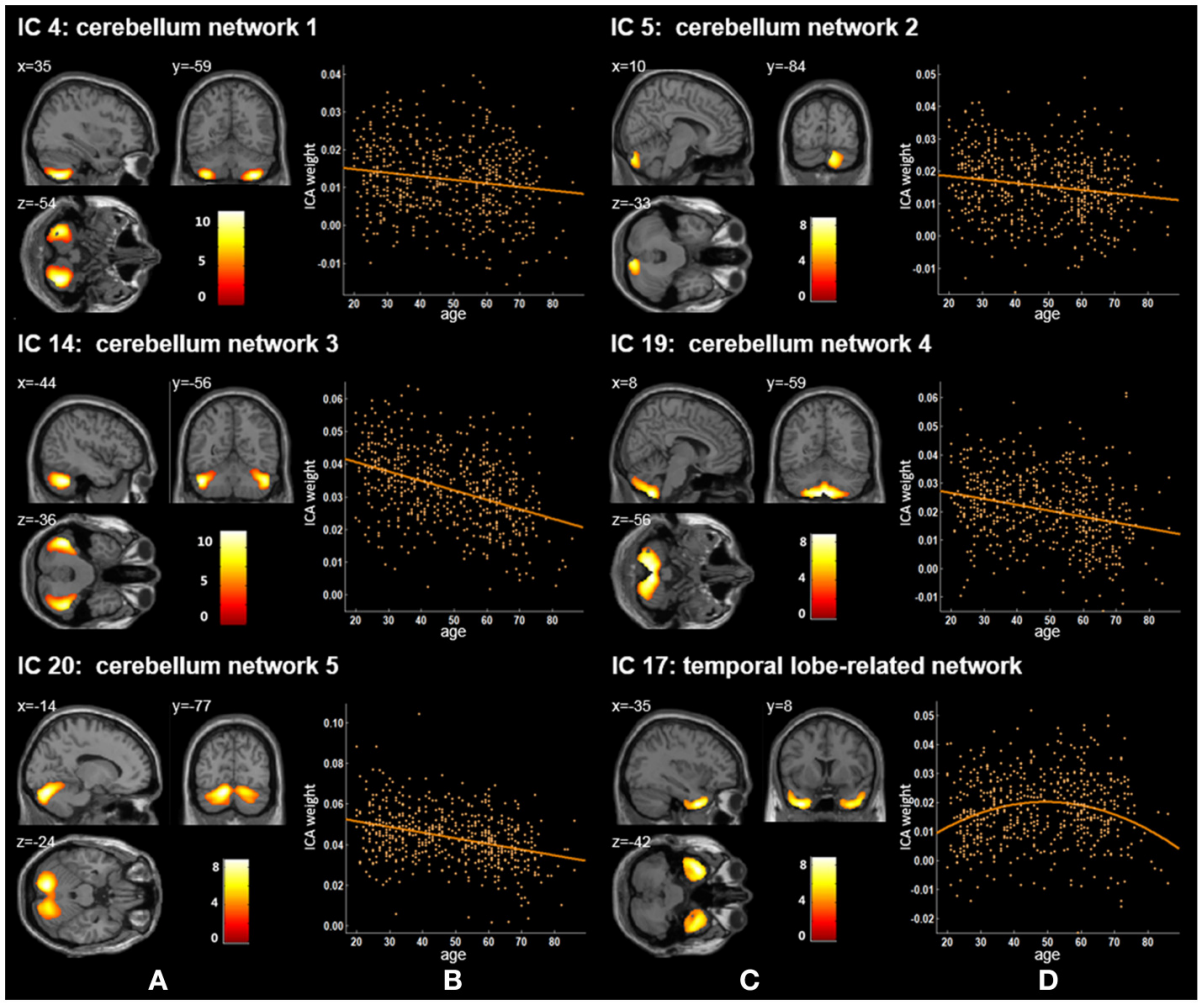
Age-related changes in gray matter structural networks. **(A)** and **(C)** columns: IC 4, 5, 14, 19, 20, and 17 represent structural network maps associated with age in 536 healthy adult subjects. The color bar represents Z scores. **(B)** and **(D)** column: the orange scatterplots show the age-related patterns in different networks. The orange lines represent the fitted lines between age and ICA weights for each network.

Twenty independent components (ICs) were extracted in the ICA. The BIC and *T*-test revealed 16 ICs significantly associated with age at Bonferroni corrected *P*-value (Figures 1–3). Fifteen ICs showed significant linear declines (*p* < 2.50E-03), and only one IC (IC 17) had a quadratic trend (*p* = 5.66E-06). These structural networks included the anterior and posterior dorsal attention networks (DAN; Figure 1, IC 2 and IC 7), the ventral and posterior DMN (Figure 1, IC 6 and IC 11), the auditory network (Figure 2, IC 12), the sensory-motor network (Figure 2, IC 15), the language-related speech network (Figure 2, IC 3), the hippocampus-related network (Figure 2, IC 16), the caudate-related network (Figure 2, IC 9), the thalamus-related network (Figure 2, IC 13), the cerebellum networks (Figure 3, IC 4, IC 5, IC 14, IC 19, and IC 20), and the temporal lobe-related network (Figure 3, IC 17). The main brain clusters in each IC are described in Table 2. The hippocampus-related network (Figure 2, IC 16) showed the most significant decreasing tendency among them (*p* = 1.11E-77).

**Table 2:** Brain regions showing age-related changes in structural networks.

**Brain regions**	**Peak coordinates**			**Z**	**Cluster size (mm^3^)**
	**MNI (X,Y,Z)**				
**IC 2: ANTERIOR DORSAL ATTENTION NETWORK**					
R middle frontal gyrus	36	27	35	10.92	11,070
L middle frontal gyrus	−35	29	32	7.93	9,140
L precentral gyrus	−38	11	47	5.77	3,139
**IC 3: LANGUAGE-RELATED SPEECH NETWORK**					
R inferior frontal gyrus	53	20	9	4.77	2,498
L inferior frontal gyrus	−51	9	15	4.5	2,929
**IC 4: CEREBELLUM NETWORK 1**					
R cerebelum 8 area	35	−59	−54	11.96	9,386
L cerebelum 8 area	−35	−56	−53	10.61	6,500
R cerebellum, lobule 7b	38	−66	−53	10.12	1,887
L cerebellum, lobule 7b	−38	−62	−53	9.44	1,684
**IC 5: CEREBELLUM NETWORK 2**					
R cerebellum crus2	12	−84	−33	6.68	3,540
R cerebellum crus1	14	−84	−30	6.36	2,005
**IC 6: VENTRAL DEFAULT MODE NETWORK**					
R middle temporal gyrus	51	−38	5	5.72	2,636
L middle temporal gyrus	−51	−41	2	8.19	8,714
R angular gyrus	45	−60	27	4.94	2,656
L angular gyrus	−42	−57	23	7.27	3,736
**IC 7: POSTERIOR DORSAL ATTENTION NETWORK**					
R postcentral gyrus	57	−20	32	8.35	8,039
L postcentral gyrus	−56	−24	30	5.87	2,015
R supramarginal gyrus	59	−18	29	8.23	5,144
L supramarginal gyrus	−53	−26	33	6.22	2,774
R inferior parietal lobule	32	−38	50	5.67	2,977
L inferior parietal lobule	−51	−27	36	6	5,528
**IC 9: CAUDATE-RELATED NETWORK**					
R caudate nucleus	14	18	0	7.43	2,626
L caudate nucleus	−12	17	−2	7.01	1,715
R putamen	18	17	0	5.22	709
L putamen	−29	3	−2	6.35	1,364
**IC 11: POSTERIOR DEFAULT MODE NETWORK**					
R precuneus	9	−50	36	7.53	8,546
L precuneus	−9	−50	35	8.89	9,383
R middle cingulate gyrus	8	−48	35	7.89	2,974
L middle cingulate gyrus	−8	−50	33	8.89	3,453
**IC 12: AUDITORY NETWORK**					
R inferior frontal gyrus	42	−27	17	7.26	2,977
R superior temporal gyrus	44	−29	17	7.12	2,504
L superior temporal gyrus	−42	−33	12	8.94	6,581
R rolandic operculum	42	−27	17	7.26	2,977
L rolandic operculum	−41	−33	14	8.81	1,826
**IC 13: THALAMUS-RELATED NETWORK**					
R thalamus	3	−18	6	9.66	3,206
L thalamus	0	−18	6	9.57	3,173
**IC 14: CEREBELLUM NETWORK 3**					
R cerebellum crus1	47	−54	−36	9.63	8,350
L cerebellum crus1	−44	−56	−36	10.68	7,212
**IC 15: SENSORY-MOTOR NETWORK**					
R superior frontal gyrus	17	6	65	6.3	6,423
L superior frontal gyrus	−23	−12	60	6.31	3,554
R middle frontal gyrus	26	14	48	4.72	1,232
L middle frontal gyrus	−27	−9	53	4.4	729
**IC 16: HIPPOCAMPUS-RELATED NETWORK**					
R hippocampus	29	−15	−21	4.08	1,033
L hippocampus	−26	−15	−21	4.19	1,205
R parahippocampal gyrus	24	−8	−24	3.97	1,073
L parahippocampal gyrus	−21	−8	−26	3.93	597
**IC 17: TEMPORAL LOBE-RELATED NETWORK**					
R inferior temporal gyrus	38	6	−42	6.14	7,243
L inferior temporal gyrus	−35	8	−42	7.52	7,459
R middle temporal pole	−35	6	−44	7.11	3,750
L middle temporal pole	45	14	−36	5.83	4,138
**IC 19: CEREBELLUM NETWORK 4**					
R cerebelum 8 area	9	−60	−56	8.9	6,247
L cerebelum 8 area	−15	−69	−53	9.04	4,604
R cerebellum crus2	15	−80	−47	6.64	4,114
L cerebellum crus2	−11	−80	−44	6.33	3,459
**IC 20: CEREBELLUM NETWORK 5**					
R cerebelum 6 area	20	−74	−24	6.23	6,281
L cerebelum 6 area	−14	−77	−24	8.68	5,947
R cerebellum crus1	20	−77	−26	5.88	4,398
L cerebellum crus1	−12	−77	−23	8.64	5,495

Figures 1–3 show age-related changes in the ICs and the corresponding scatterplots with best fitted curves between ICA weights and age for each IC. Table 3 lists the results of the regression statistics analysis.

**Table 3:** *T*-test for regression analyses of ICA weights with age in different ICs.

**Network**	**Regression statistics**			
	***R*^2^**	Beta^*^ (95% confidence intervals)	***t***	***P***
IC 2: anterior dorsal attention network	0.246	−3.75E-04 (−4.31E-004, −3.19E-04)	−13.19	1.36E-34
IC 3: language-related speech network	0.139	−3.87E-04 (−4.69E-04, −3.05E-04)	−9.29	3.77E-19
IC 4: cerebellum network 1	0.025	−9.44E-05 (−1.45E-04, −4.41E-05)	−3.68	2.54E-04
IC 5: cerebellum network 2	0.028	−1.07E-04 (−1.61E-04, −5.36E-05)	−3.93	9.80E-05
IC 6: ventral default mode network	0.340	−4.97E-04 (−5.56E-04, −4.38E-04)	−16.57	4.66E-50
IC 7: posterior dorsal attention network	0.096	−2.29E-04 (−2.89E-04, −1.70E-04)	−7.52	2.40E-13
IC 9: caudate-related network	0.168	−3.14E-04 (−3.73E-04, −2.54E-04)	−10.37	4.35E-23
IC 11: posterior default mode network	0.043	−1.46E-04 (−2.04E-04, −8.71E-05)	−4.88	1.41E-06
IC 12: auditory network	0.306	−4.58E-04 (−5.16E-04, −3.99E-04)	−15.34	2.90E-44
IC 13: thalamus-related network	0.113	−3.92E-04 (−4.85E-04, −2.98E-04)	−8.24	1.32E-15
IC 14: cerebellum network 3	0.157	−2.89E-04 (−3.45E-04, −2.32E-04)	−9.96	1.56E-21
IC 15: sensory-motor network	0.280	−4.96E-04 (−5.64E-04, −4.29E-04)	−14.41	5.44E-40
IC 16: hippocampus-related network	0.479	−5.67E-04 (−6.17E-04, −5.17E-04)	−22.17	1.11E-77
IC 17: temporal lobe-related network	0.039	−1.01E-05 (−1.44 E-05, −5.75 E-06)	−4.59	5.66E-06
IC 19: cerebellum network 4	0.070	−2.09E-04 (−2.73E-04, −1.44E-04)	−6.36	4.47E-10
IC 20: cerebellum network 5	0.118	−2.80E-04 (−3.45E-04, −2.15E-04)	−8.45	2.88E-16

* *Beta of the highest item in the regression model*.

The results for subjects aged 20–80 years showed that the ICA weights of the same 15 ICs exhibited significant linear declines (*p* < 2.50E-03), and only that of IC 17 had a significant quadratic trend (*p* = 4.17E-04). The results for subjects aged 20–70 years showed that there were still the same 14 ICs showing significant linear declines (*p* < 2.50E-03), but one IC (IC 4) showed non-significant linear reduce (*p* = 0.0191). In addition, IC 17 was also had a quadratic trend with non-significant level (*p* of age^2^ = 0.0081). The results for subjects aged 20–60 years showed that all 16 ICs had the linear decreased patterns. Twelve of 16 ICs were still significant (*p* < 2.50E-03) and the remaining 4 ICs (IC 4, 17, 19, 20) were non-significant with a minimum of *p* = 0.0062.

## Discussion

In the current study, we first performed multivariate ICA to investigate the brain structural covariance networks across the adult lifespan based on healthy subjects' MRI data acquired from the dataset IXI. Then, we further explored the trajectories of the structural networks associated with age. We found 16 significant age-related networks, and ICA weights of 15 networks decreased linearly with age; only ICA weights of the temporal lobe-related network (IC 17) showed a significant quadratic tendency with age.

In previous studies, researchers have extracted the DAN based on functional MRI (fMRI) data (De Luca et al., 2006; Fox et al., 2006; Mantini et al., 2007; Power et al., 2011). For example, Mantini et al. decomposed fMRI data via ICA to investigate the brain resting state networks from 15 healthy subjects (20–29 years) and obtained a DAN network mainly including the bilateral intraparietal sulcus (Mantini et al., 2007). We reported that IC 2 and IC 7 corresponded to the anterior and posterior DAN. Some resting state functional studies have explored functional connectivity density (FCD) changes of DAN across lifespan (Tomasi and Volkow, 2012; Betzel et al., 2014). Betzel et al. found two dorsal attention components (DorsAttnA and DorsAttnB), and the modularity of DorsAttnA which mainly located to the temporo-occipital cortex, parieto-occipital cortex, and superior parietal lobule showed a prominent age-related linear decrease of FCD across the subjects aged 7–85 years (Betzel et al., 2014). Tomasi and Volkow used a FCD mapping approach and revealed statistically significant age-related FCD decreases in DAN (*r* = −0.23, *p* < 1.00E-06) from healthy subjects (13–85 years) (Tomasi and Volkow, 2012). Our current results showed that gray matter volumes of IC 2 and IC 7 also exhibited significant linearly decreased trends with age (*p* = 1.36E-34 and *p* = 2.40E-13, respectively), which suggested that functional and structural DAN have similar age-related patterns.

We found that ICA weights of both the ventral and posterior DMN (IC 6 and IC 11) declined linearly with age ranging from 20 to 86 (Figure 1, Tables 2, 3). Several neuroimaging studies have proposed that the structural DMN changes with age not only during the developmental process (Bluhm et al., 2008; Supekar et al., 2010) but also in adult life (Luo et al., 2012; Spreng and Turner, 2013; Hafkemeijer et al., 2014). Spreng et al. suggested a significant linear decline between age and the structural covariance of the default network scores across the adult lifespan of 18–96 years (Spreng and Turner, 2013). Meanwhile, Hafkemeijer et al. revealed that there was a negative association between age and gray matter volume in the DMN from 45 to 85 years of age (Hafkemeijer et al., 2014), in agreement with our results. Moreover, age-related changes can also be found in the functional connectivity (FC) in the DMN (Damoiseaux et al., 2008; Hafkemeijer et al., 2012; Onoda et al., 2012; Huang et al., 2015). Damoiseaux et al. demonstrated that the FC of the DMN decreased in older participants (age 70.7 ± 6.0 years) relative to young participants (age 22.8 ± 2.3 years) (Damoiseaux et al., 2008). The DMN consists of sub-networks and different sub-networks are responsible for different cognitive functions (Uddin et al., 2009; Damoiseaux et al., 2012; Huang et al., 2015). The degree to which age affects the relevant cognitive functions of default mode sub-networks seems to be different (Huang et al., 2015). We also demonstrated that these two DMN ICs presented significant declining trends but with different degrees (*p* = 4.66E-50 for the ventral DMN and *p* = 1.41E-06 for the posterior DMN), possibly because of DMN sub-network's distinct cognitive functions. When considered together, these findings indicate that the decreased functional connectivity within the DMN may be associated with structural network changes of the DMN.

Our ICA results found three other gray matter covariant networks: the auditory network (IC 12), the sensory-motor network (IC 15), and the language-related speech network (IC 3; Figure 1 and Table 2). Significant linear decrease trajectories were found between the ICA weights and age in these three networks (Table 3). Li et al. also found that the structural associations in the auditory network and language-related speech network decreased significantly with age between the young and middle-aged groups and were relatively preserved or mildly changed between the middle-aged and old groups (Li et al., 2013). Whereas, they found that there was an increased tendency in structural associations within the motor network from the young group (18–23 years) to the middle-aged group (30–58 years) which was different from ours, and a downtrend from the middle-aged to the old group (60–69 years) but no significant difference between the young and old groups (Li et al., 2013). In addition, Zielinski et al. investigated the developmental structural changes in these networks based on children and adolescents in four age categories (from 5 to 18 years) and found that the primary auditory and motor networks largely developed in early adolescence; in contrast, the language-related speech network showed a significant expansion in late adolescence (Zielinski et al., 2010). Further, an accelerated decline on the gray matter volume in the middle and superior frontal gyrus (the main brain areas of IC 15) in ages older than 20 years was reported (Giorgio et al., 2010). A number of studies have illustrated an accelerated loss of gray matter volume in auditory-related regions (the main brain areas of IC 12) in aging adult brains (Good et al., 2001; Lemaître et al., 2005; Kalpouzos et al., 2009). The significant decline trends from 20 to 86 years old showed by IC 12 and IC 15 in our study are consistent with the regional patterns of age-related gray matter loss in these studies.

Apart from the ICs discussed above, IC 16 included the left and right hippocampus and parahippocampal gyrus (Figure 2) and showed the most significant decreasing tendency among all ICs (*p* = 1.11E-77). Several studies have consistently reported an accelerated decline of the gray matter volume in the hippocampus with age (Manrique et al., 2009; Fjell et al., 2013). Fjell et al. delineated age-related trajectories of the volume of 17 ROIs in healthy adults (18–94 years) via a non-parametric smoothing spline approach, and the hippocampus showed the fastest loss rate (Fjell et al., 2013). Although, we employed different method from Fjell et al., nevertheless, the gray matter volume of hippocampus-related network also had the most severe aging-related atrophy (*p* = 1.11E-77) in comparison to those of other networks. Further, the decline of memory and cognitive abilities with age has been frequently discussed (Schönknecht et al., 2005; Manrique et al., 2009; Rosenbaum et al., 2015). Our findings add to the growing evidence that memory deficits of aging may be related to the atrophy of the hippocampus.

We also identified five cerebellum networks: IC 4, IC 5, IC 14, IC 19, and IC 20 (Figure 2 and Table 2). O'Reilly et al. found that cerebellum contained at least two zones, including a primary sensorimotor zone and a supramodal zone, which were equivalent to our network IC 14 (O'reilly et al., 2010). Dobromyslin et al. even found multiple cerebellar networks, and four of our cerebellum networks (except IC 5) were spatially similar to four of theirs (except a, f, g) (Dobromyslin et al., 2012). Significant linear declines between ICA weights and age were observed in these five cerebellum networks (Table 3). Raz et al. revealed an age-related linear decline in the volume of cerebellar hemispheres and vermis based on healthy adults aged 18–81 years, which is in agreement with the trajectory of our cerebellum networks (Raz et al., 2001). The cerebellum is commonly involved in motor coordination and now is also considered to be related to the modulation of cognition and learning (Raz et al., 2000; Bernard and Seidler, 2014).

In our results, the trajectory of the temporal lobe-related network (IC 17; Figure 2 and Table 2), a quadratic decrease over age (*t* = −4.59, *p* of age^2^ = 5.66E-06), showed an increasing trend from 20 to 50 years old and was followed by an obvious decline. Previously, age-related differences of the temporal anatomical network have been reported (Alexander et al., 2006; Brickman et al., 2007; Douaud et al., 2014; Hafkemeijer et al., 2014). Hafkemeijer et al. found that the temporal lobe-related network (network e) showed the decreased trend with non-significant level in middle-aged to older adults (Hafkemeijer et al., 2014). Douaud et al. assessed brain structure networks among normal subjects (8–85 years) and the brain network mainly including the medial temporal areas showed a symmetric and strong non-monotonic relationship with age (Douaud et al., 2014). Alexander et al. used the SSM method to identify structural network patterns associated with age from healthy adults (22–77 years) and found older age was associated with less gray matter in the frontal and temporal brain regions (Alexander et al., 2006). Sowell et al. found that the gray matter density in the temporal-related area showed a non-linear change with an inverted U-shaped curve with age across the lifespan (7 to 87 years) (Sowell et al., 2003). Because of the late maturation pattern of the temporal lobe in the human brain (Gogtay et al., 2004) and the memory, recognition and other functions related to the temporal lobe (Macsweeney et al., 2002; Diaconescu et al., 2013; Perrodin et al., 2014), the temporal lobe-related network might mature after other brain areas, followed by atrophy, thus presenting an inverse U-shaped tendency with age.

For two subcortical structures, IC 9 was recognized as a caudate-related network, and IC 13 recognized as a thalamus-related network. Damoiseaux et al. also found that the functional brain network K, which contains the thalamus, putamen, insular, and transverse temporal gyrus, showed spatial overlap with IC 13 in this study (Damoiseaux et al., 2008). The thalamus-related network's path in our results showed a significant linear reduction across the adult stage (Figure 2 and Table 3), in accordance with Hafkemeijer et al.'s study, in which the gray matter volume in this network displayed a slightly negative association with age (Hafkemeijer et al., 2014). Although, other studies reported age-related neuroanatomical volume changes in subcortical structures, such as the caudate and thalamus, their findings were not exactly consistent (Fjell et al., 2013; Pfefferbaum et al., 2013; Serbruyns et al., 2015). Serbruyns et al. have investigated the subregional atrophy of bilateral thalamus and caudate from 22 to 79 years old, and the right thalamus showed atrophy from the 5th decade, while 6th decade for the left thalamus and the bilateral caudate (Serbruyns et al., 2015). In Pfefferbaum et al.'s study, the best fitted trajectories of the thalamus and caudate from 20 to 85 years were quadratic models (Pfefferbaum et al., 2013). Till now, there are relatively few studies on these two networks. Thus, their network patterns associated with age need to be investigated further.

We re-performed the same statistical analysis of the ICs for three different age group (20–80, 20–70, and 20–60 years) to evaluate the age range effect on the age-related patterns. Compared with significant age-related results for subjects aged 20–86 years, the results for subjects aged 20–80 years showed the same age-related patterns. Though these results had different *p*-values from the original analysis, all met the significance level. The results for subjects aged 20–70 years showed the similar age-related patterns. To be specific, among the reported 15 linear ICs, there were still the same significant 14 ICs, but one non-significant IC (IC 4). In addition, IC 17 was also had a quadratic trend with non-significant level. The results for subjects aged 20–60 years showed the slightly different age-related patterns. Specifically, all 16 ICs showed the linear decreased trends with significant 12 ICs and four non-significant ICs (IC 4, 17, 19, 20). Overall, the results of these additional analyses are consistent with our original findings and also demonstrated reasonably the age range effect on age-related change patterns of brain structural networks. In our results of subjects aged 20–86 years, the trajectory of the IC 17 showed a quadratic change over age. However, for a shorter age range, such as 20–70 and 20–60 years, a quadratic trend over age was not so obvious or even changed to be a linear path. Indeed, the significance level is associated with the sample size and the age distribution of participants in each age group. We should report the results with caution and clearly declare the age-related patterns with the specific subject age range.

A specific limitation of our study is the estimation of the number of components. For ICA-related studies, there is a lack of available standards to determine the optimal number of ICA components. Most studies adopted 12 to 25 components in structural networks or resting-state functional networks (Beckmann et al., 2005; Damoiseaux et al., 2006, 2008; Smith et al., 2009). Based on these studies, we chose 20 as the ICA output number. Additionally, the number of available subjects aged more than 80 years was relatively smaller than those of other age groups in the Information IXI database (http://www.brain-development.org). More subjects older than 80 years needed to be included to confirm our findings. Finally, our study only investigated structural MRI data and lacked resting-state fMRI and diffusion tensor imaging (DTI) data. In future studies, we shall combine multi-modality data to examine anatomical and functional networks and the age-related relationships between them.

## Conclusion

In the current study, we used a multivariate ICA method to investigate the structural covariance patterns of gray matter volume through adulthood in 536 healthy subjects. Sixteen structural networks, with the exception of the temporal lobe-related network, showed a linear decline trajectory with age from 20 to 86 years. Our results largely confirmed previously reported findings. We noticed the confirmatory nature of our findings but for continuous age range so further extending the previous findings. Our findings not only provide insight into the patterns of age-related structural changes based on the network in the human brain, but also provide a foundation for understanding abnormal aging.

## Author contributions

KLiu, SY, KC, LY, and XG conceived and designed the study. KLiu, SY, KC, and XG developed the methods. KLiu and SY analyzed and interpreted data. JZ, LY, KLi, and ZJ interpreted data. KLiu, SY, and XG drafted the manuscript.

### Conflict of interest statement

The authors declare that the research was conducted in the absence of any commercial or financial relationships that could be construed as a potential conflict of interest.

## References

[B1] Alexander G. E. Chen K. Merkley T. L. Reiman E. M. Caselli R. J. Aschenbrenner M.. (2006). Regional network of magnetic resonance imaging gray matter volume in healthy aging. Neuroreport 17, 951–956. 10.1097/01.wnr.0000220135.16844.b6 16791083

[B2] Allen J. S. Bruss J. Brown C. K. Damasio H. (2005). Normal neuroanatomical variation due to age: the major lobes and a parcellation of the temporal region. Neurobiol. Aging 26, 1245–1260. 10.1016/j.neurobiolaging.2005.05.023 16046030

[B3] Ashburner J. (2007). A fast diffeomorphic image registration algorithm. Neuroimage 38, 95–113. 10.1016/j.neuroimage.2007.07.007 17761438

[B4] Ashburner J. Friston K. J. (2000). Voxel-based morphometry—the methods. Neuroimage 11, 805–821. 10.1006/nimg.2000.0582 10860804

[B5] Beckmann C. F. Deluca M. Devlin J. T. Smith S. M. (2005). Investigations into resting-state connectivity using independent component analysis. Philos. Trans. R. Soc. Lond. B. Biol. Sci. 360, 1001–1013. 10.1098/rstb.2005.1634 16087444 PMC1854918

[B6] Bergfield K. L. Hanson K. D. Chen K. Teipel S. J. Hampel H. Rapoport S. I.. (2010). Age-related networks of regional covariance in MRI gray matter: reproducible multivariate patterns in healthy aging. Neuroimage 49, 1750–1759. 10.1016/j.neuroimage.2009.09.051 19796692 PMC2789892

[B7] Bernard J. A. Seidler R. D. (2014). Moving forward: age effects on the cerebellum underlie cognitive and motor declines. Neurosci. Biobehav. Rev. 42, 193–207. 10.1016/j.neubiorev.2014.02.011 24594194 PMC4024443

[B8] Betzel R. F. Byrge L. He Y. Goñi J. Zuo X.-N. Sporns O. (2014). Changes in structural and functional connectivity among resting-state networks across the human lifespan. Neuroimage 102, 345–357. 10.1016/j.neuroimage.2014.07.067 25109530

[B9] Bluhm R. L. Osuch E. A. Lanius R. A. Boksman K. Neufeld R. W. Théberge J.. (2008). Default mode network connectivity: effects of age, sex, and analytic approach. Neuroreport 19, 887–891. 10.1097/WNR.0b013e328300ebbf 18463507

[B10] Brickman A. M. Habeck C. Zarahn E. Flynn J. Stern Y. (2007). Structural MRI covariance patterns associated with normal aging and neuropsychological functioning. Neurobiol. Aging 28, 284–295. 10.1016/j.neurobiolaging.2005.12.016 16469419

[B11] Calhoun V. D. Liu J. Adalı T. (2009). A review of group ICA for fMRI data and ICA for joint inference of imaging, genetic, and ERP data. Neuroimage 45, S163–S172. 10.1016/j.neuroimage.2008.10.057 19059344 PMC2651152

[B12] Curiati P. Tamashiro J. Squarzoni P. Duran F. Santos L. Wajngarten M.. (2009). Brain structural variability due to aging and gender in cognitively healthy Elders: results from the Sao Paulo Ageing and Health study. Am. J. Neuroradiol. 30, 1850–1856. 10.3174/ajnr.A1727 19661175 PMC7051289

[B13] Damoiseaux J. S. Beckmann C. F. Arigita E. J. Barkhof F. Scheltens P. Stam C. J.. (2008). Reduced resting-state brain activity in the “default network” in normal aging. Cereb. Cortex 18, 1856–1864. 10.1093/cercor/bhm207 18063564

[B14] Damoiseaux P. K. E. Miller B. L. Greicius M. D. (2012). Functional connectivity tracks clinical deterioration in Alzheimer's disease. Neurobiol. Aging 33, 828.e19–e828.e30. 10.1016/j.neurobiolaging.2011.06.024 21840627 PMC3218226

[B15] Damoiseaux R. S. A. Barkhof F. Scheltens P. Stam C. J. Smith S. M. Beckmann C. F. (2006). Consistent resting-state networks across healthy subjects. Proc. Natl. Acad. Sci. U.S.A. 103, 13848–13853. 10.1073/pnas.0601417103 16945915 PMC1564249

[B16] De Luca M. Beckmann C. F. De Stefano N. Matthews P. M. Smith S. M. (2006). fMRI resting state networks define distinct modes of long-distance interactions in the human brain. Neuroimage 29, 1359–1367. 10.1016/j.neuroimage.2005.08.035 16260155

[B17] Diaconescu A. O. Hasher L. McIntosh A. R. (2013). Visual dominance and multisensory integration changes with age. Neuroimage 65, 152–166. 10.1016/j.neuroimage.2012.09.057 23036447

[B18] Dobromyslin V. I. Salat D. H. Fortier C. B. Leritz E. C. Beckmann C. F. Milberg W. P.. (2012). Distinct functional networks within the cerebellum and their relation to cortical systems assessed with independent component analysis. Neuroimage 60, 2073–2085. 10.1016/j.neuroimage.2012.01.139 22342804 PMC3549335

[B19] Douaud G. Groves A. R. Tamnes C. K. Westlye L. T. Duff E. P. Engvig A.. (2014). A common brain network links development, aging, and vulnerability to disease. Proc. Natl. Acad. Sci. 111, 17648–17653. 10.1073/pnas.1410378111 25422429 PMC4267352

[B20] Fjell A. M. Westlye L. T. Grydeland H. Amlien I. Espeseth T. Reinvang I.. (2013). Critical ages in the life course of the adult brain: nonlinear subcortical aging. Neurobiol. Aging 34, 2239–2247. 10.1016/j.neurobiolaging.2013.04.006 23643484 PMC3706494

[B21] Fox M. D. Corbetta M. Snyder A. Z. Vincent J. L. Raichle M. E. (2006). Spontaneous neuronal activity distinguishes human dorsal and ventral attention systems. Proc. Natl. Acad. Sci. U.S.A. 103, 10046–10051. 10.1073/pnas.0604187103 16788060 PMC1480402

[B22] Ge Y. Grossman R. I. Babb J. S. Rabin M. L. Mannon L. J. Kolson D. L. (2002). Age-related total gray matter and white matter changes in normal adult brain. Part I: volumetric MR imaging analysis. Am. J. Neuroradiol. 23, 1327–1333. 12223373 PMC7976241

[B23] Giorgio A. Santelli L. Tomassini V. Bosnell R. Smith S. De Stefano N.. (2010). Age-related changes in grey and white matter structure throughout adulthood. Neuroimage 51, 943–951. 10.1016/j.neuroimage.2010.03.004 20211265 PMC2896477

[B24] Gogtay N. Giedd J. N. Lusk L. Hayashi K. M. Greenstein D. Vaituzis A. C.. (2004). Dynamic mapping of human cortical development during childhood through early adulthood. Proc. Natl. Acad. Sci. U.S.A. 101, 8174–8179. 10.1073/pnas.0402680101 15148381 PMC419576

[B25] Good C. Johnsrude I. Ashburner J. Henson R. Friston K. Frackowiak R. (2001). A voxel-based morphometric study of ageing in 465 normal adult human brains. Neuroimage 14, 21–36. 10.1006/nimg.2001.0786 11525331

[B26] Guo X. Wang Y. Guo T. Chen K. Zhang J. Li K.. (2014). Structural covariance networks across healthy young adults and their consistency. J. Magn. Reson. Imaging 42, 261–268. 10.1002/jmri.24780 25327998

[B27] Hafkemeijer A. Altmann-Schneider I. De Craen A. Slagboom P. Van Der Grond J. Rombouts S. (2014). Associations between age and gray matter volume in anatomical brain networks in middle-aged to older adults. Aging Cell 13, 1068. 10.1111/acel.12271 25257192 PMC4326918

[B28] Hafkemeijer A. Van Der Grond J. Rombouts S. (2012). Imaging the default mode network in aging and dementia. Biochim. Biophys. Acta 1822, 431–441. 10.1016/j.bbadis.2011.07.008 21807094

[B29] Huang C. C. Hsieh W. J. Lee P. L. Peng L. N. Liu L. K. Lee W. J.. (2015). Age-related changes in resting-state networks of a large sample size of healthy elderly. CNS Neurosci. Ther. 21, 817–825. 10.1111/cns.12396 25864728 PMC6493082

[B30] Kalpouzos G. Chetelat G. Baron J. C. Landeau B. Mevel K. Godeau C.. (2009). Voxel-based mapping of brain gray matter volume and glucose metabolism profiles in normal aging. Neurobiol. Aging 30, 112–124. 10.1016/j.neurobiolaging.2007.05.019 17630048

[B31] Kennedy D. N. Haselgrove C. Riehl J. Preuss N. Buccigrossi R. (2016). The NITRC image repository. Neuroimage 124, 1069–1073. 10.1016/j.neuroimage.2015.05.074 26044860 PMC4651733

[B32] Lemaître H. Crivello F. Grassiot B. Alpérovitch A. Tzourio C. Mazoyer B. (2005). Age-and sex-related effects on the neuroanatomy of healthy elderly. Neuroimage 26, 900–911. 10.1016/j.neuroimage.2005.02.042 15955500

[B33] Li X. Pu F. Fan Y. Niu H. Li S. Li D. (2013). Age-related changes in brain structural covariance networks. Front. Hum. Neurosci. 7, 7–98. 10.3389/fnhum.2013.00098 23532684 PMC3607831

[B34] Luo L. Xu L. Jung R. Pearlson G. Adali T. Calhoun V. D. (2012). Constrained source-based morphometry identifies structural networks associated with default mode network. Brain Connect. 2, 33–43. 10.1089/brain.2011.0026 22468608 PMC3621809

[B35] Macsweeney M. Woll B. Campbell R. McGuire P. K. David A. S. Williams S. C.. (2002). Neural systems underlying British Sign Language and audio-visual English processing in native users. Brain 125, 1583–1593. 10.1093/brain/awf153 12077007

[B36] Manrique T. Morón I. Ballesteros M. A. Guerrero R. M. Fenton A. A. Gallo M. (2009). Hippocampus, aging, and segregating memories. Hippocampus 19, 57–65. 10.1002/hipo.20481 18680140

[B37] Mantini D. Perrucci M. G. Del Gratta C. Romani G. L. Corbetta M. (2007). Electrophysiological signatures of resting state networks in the human brain. Proc. Natl. Acad. Sci. U.S.A. 104, 13170–13175. 10.1073/pnas.0700668104 17670949 PMC1941820

[B38] McIntosh A. R. Mišic B. (2013). Multivariate statistical analyses for neuroimaging data. Annu. Rev. Psychol. 64, 499–525. 10.1146/annurev-psych-113011-143804 22804773

[B39] Mills K. L. Lalonde F. Clasen L. S. Giedd J. N. Blakemore S.-J. (2014). Developmental changes in the structure of the social brain in late childhood and adolescence. Soc. Cogn. Affect. Neurosci. 9, 123–131. 10.1093/scan/nss113 23051898 PMC3871734

[B40] Onoda K. Ishihara M. Yamaguchi S. (2012). Decreased functional connectivity by aging is associated with cognitive decline. J. Cogn. Neurosci. 24, 2186–2198. 10.1162/jocn_a_00269 22784277

[B41] O'reilly J. X. Beckmann C. F. Tomassini V. Ramnani N. Johansen-Berg H. (2010). Distinct and overlapping functional zones in the cerebellum defined by resting state functional connectivity. Cereb. Cortex 20, 953–965. 10.1093/cercor/bhp157 19684249 PMC2837094

[B42] Perrodin C. Kayser C. Logothetis N. K. Petkov C. I. (2014). Auditory and visual modulation of temporal lobe neurons in voice-sensitive and association cortices. J. Neurosci. 34, 2524–2537. 10.1523/JNEUROSCI.2805-13.2014 24523543 PMC3921424

[B43] Pfefferbaum A. Rohlfing T. Rosenbloom M. J. Chu W. Colrain I. M. Sullivan E. V. (2013). Variation in longitudinal trajectories of regional brain volumes of healthy men and women (ages 10 to 85 years) measured with atlas-based parcellation of MRI. Neuroimage 65, 176–193. 10.1016/j.neuroimage.2012.10.008 23063452 PMC3516371

[B44] Power J. D. Cohen A. L. Nelson S. M. Wig G. S. Barnes K. A. Church J. A.. (2011). Functional network organization of the human brain. Neuron 72, 665–678. 10.1016/j.neuron.2011.09.006 22099467 PMC3222858

[B45] Raji C. A. Lopez O. L. Kuller L. H. Carmichael O. T. Longstreth W. T. Gach H. M.. (2012). White matter lesions and brain gray matter volume in cognitively normal elders. Neurobiol. Aging 33, 834.e7–834.e16. 10.1016/j.neurobiolaging.2011.08.010 21943959 PMC3248984

[B46] Raz N. Gunning-Dixon F. Head D. Williamson A. Acker J. D. (2001). Age and sex differences in the cerebellum and the ventral pons: a prospective MR study of healthy adults. Am. J. Neuroradiol. 22, 1161–1167. 11415913 PMC7974784

[B47] Raz N. Williamson A. Gunning-Dixon F. Head D. Acker J. D. (2000). Neuroanatomical and cognitive correlates of adult age differences in acquisition of a perceptual-motor skill. Microsc. Res. Tech. 51, 85–93. 10.1002/1097-0029(20001001)51:1<85::AID-JEMT9>3.0.CO;2-0 11002356

[B48] Rosenbaum R. S. Winocur G. Binns M. A. Moscovitch M. (2015). Remote spatial memory in aging: all is not lost. Front. Aging Neurosci. 4:25 10.3389/fnagi.2012.00025 PMC3440628 22993506

[B49] Schönknecht P. Pantel J. Kruse A. Schröder J. (2005). Prevalence and natural course of aging-associated cognitive decline in a population-based sample of young-old subjects. Am. J. Psychiatry 162, 2071–2077. 10.1176/appi.ajp.162.11.2071 16263846

[B50] Serbruyns L. Leunissen I. Huysmans T. Cuypers K. Meesen R. L. Van Ruitenbeek P.. (2015). Subcortical volumetric changes across the adult lifespan: subregional thalamic atrophy accounts for age-related sensorimotor performance declines. Cortex 65, 128–138. 10.1016/j.cortex.2015.01.003 25682047

[B51] Smith S. M. Fox P. T. Miller K. L. Glahn D. C. Fox P. M. Mackay C. E.. (2009). Correspondence of the brain's functional architecture during activation and rest. Proc. Natl. Acad. Sci. U.S.A. 106, 13040–13045. 10.1073/pnas.0905267106 19620724 PMC2722273

[B52] Sowell E. R. Peterson B. S. Thompson P. M. Welcome S. E. Henkenius A. L. Toga A. W. (2003). Mapping cortical change across the human life span. Nat. Neurosci. 6, 309–315. 10.1038/nn1008 12548289

[B53] Spreng R. N. Turner G. R. (2013). Structural covariance of the default network in healthy and pathological aging. J. Neurosci. 33, 15226–15234. 10.1523/JNEUROSCI.2261-13.2013 24048852 PMC3776065

[B54] Supekar K. Uddin L. Q. Prater K. Amin H. Greicius M. D. Menon V. (2010). Development of functional and structural connectivity within the default mode network in young children. Neuroimage 52, 290–301. 10.1016/j.neuroimage.2010.04.009 20385244 PMC2976600

[B55] Tomasi D. Volkow N. D. (2012). Aging and functional brain networks. Mol. Psychiatry 17, 549–558. 10.1038/mp.2011.81 21727896 PMC3193908

[B56] Uddin L. Q. Clare Kelly A. Biswal B. B. Xavier Castellanos F. Milham M. P. (2009). Functional connectivity of default mode network components: correlation, anticorrelation, and causality. Hum. Brain Mapp. 30, 625–637. 10.1002/hbm.20531 18219617 PMC3654104

[B57] Xu L. Groth K. M. Pearlson G. Schretlen D. J. Calhoun V. D. (2009). Source-based morphometry: the use of independent component analysis to identify gray matter differences with application to schizophrenia. Hum. Brain Mapp. 30, 711–724. 10.1002/hbm.20540 18266214 PMC2751641

[B58] Ziegler G. Dahnke R. Jäncke L. Yotter R. A. May A. Gaser C. (2012). Brain structural trajectories over the adult lifespan. Hum. Brain Mapp. 33, 2377–2389. 10.1002/hbm.21374 21898677 PMC6870331

[B59] Zielinski B. A. Gennatas E. D. Zhou J. Seeley W. W. (2010). Network-level structural covariance in the developing brain. Proc. Natl. Acad. Sci. U.S.A. 107, 18191–18196. 10.1073/pnas.1003109107 20921389 PMC2964249

